# Serum apolipoprotein B to apolipoprotein A-I ratio is an independent predictor of liver metastasis from locally advanced rectal cancer in patients receiving neoadjuvant chemoradiotherapy plus surgery

**DOI:** 10.1186/s12885-021-09101-y

**Published:** 2022-01-03

**Authors:** Chen Chen, Wei Yi, Zhi-fan Zeng, Qiao-xuan Wang, Wu Jiang, Yuan-hong Gao, Hui Chang

**Affiliations:** 1 Department of Radiation Oncology, Sun Yat-sen University Cancer Center, Guangzhou, China; 2 State Key Laboratory of Oncology in South China, Collaborative Innovation Center for Cancer Medicine, Guangzhou, China; 3 Department of Radiation Oncology, the First Affiliated Hospital of Guangzhou Medical University, Guangzhou, China; 4 Department of Colorectal Surgery, Sun Yat-sen University Cancer Center, Guangzhou, China

**Keywords:** Rectal cancer, Liver metastasis, Apolipoprotein, Survival

## Abstract

**Background:**

The ratio of serum apolipoprotein B (apoB) to apolipoprotein A-I (apoAI) had been reported as a prognostic factor in colorectal cancer. This retrospective study aimed to assess the implication of apoB-to-apoAI ratio in predicting liver metastasis from rectal cancer (RC).

**Methods:**

The clinical data of 599 locally advanced RC patients treated with chemoradiotherapy followed by surgery were reviewed. Serum apoAI, apoB and apoB-to-apoAI ratio were analyzed for their correlation with the liver-metastasis-free, other-metastasis-free and overall survivals, together with the pretreatment and postsurgical pathoclinical features of the patients. Univariate and multivariate survival analyses were realized through the Kaplan-Meier approach and Cox model, respectively. Hazard ratios (HRs) and 95% confidence intervals (CIs) were calculated for independent predictors.

**Results:**

Carbohydrate antigen 19 − 9 ≥ 26.3 U/ml, apoB-to-apoAI ratio ≥ 0.63, tumor regression grade 5 − 3, pT4 and pN + stage emerged as independent predictors of poorer liver-metastasis-free survival. The hazard ratios were 1.656 (95% CI, 1.094–2.506), 1.919 (95% CI, 1.174–3.145), 1.686 (95% CI, 1.053–2.703), 1.890 (95% CI, 1.110–3.226) and 2.012 (95% CI, 1.314–2.077), respectively. Except apoB-to-apoAI ratio, the other 4 factors were also independent predictors of poorer other-metastasis-free and overall survivals. And the independent predictors of poorer overall survival also included age ≥ 67 years old, distance to anal verge < 5 cm.

**Conclusions:**

Serum apoB-to-apoAI ratio could be used as a biomarker for prediction of liver metastasis risk in locally advanced RC.

**Supplementary Information:**

The online version contains supplementary material available at 10.1186/s12885-021-09101-y.

## Background

Currently, locally advanced rectal cancer (RC) is primarily treated by chemoradiotherapy plus surgery [1]. Despite an ideal local control, the 5-year distant metastasis rate remains to be more than 20% [2]. Based on published studies, liver metastasis (LM) is the most common type of distant failure and leads to a median survival time of merely 19.7 months. Yet, the figure could rise to 64.5 months when the LM lesion is resectable [3, 4]. For improving patient survival, it is important to predict LM or diagnose it at a resectable size.

Until now, few factors are identified for specific prediction of LM from RC. In a study by Chen et al., commonly used anatomical prognosticators of RC were analyzed for their association with LM. Of those, only distal tumor appeared to predict a higher LM risk [5]. Serum biomarkers could indirectly reflect tumor-host interactions and be considered as candidate predictors of LM. Meltzer et al. reported that elevated circulating sCD40 level correlated independently with a shorter time to LM [6]. But the analysis procedure of sCD40 was complicated and needed advanced examination conditions. Thus, there is a need to find predictors of LM from routinely tested indices.

Apolipoprotein is a family of serum proteins which facilitate lipid transportation and are discovered recently to participate in cancer metabolism and immunity [7]. Some apolipoproteins have exhibited their prognostic values in colorectal cancer. The serum level of apolipoprotein A-I (apoAI) and apolipoprotein B (apoB) were reported as positive and negative predictors of patient survival, respectively [8–10]. Yang et al. further combined these 2 indices into apoB-to-apoAI ratio and achieved improved prediction efficiency [11]. In the literature, the values of apoAI and apoB in predicting LM of RC are still unknown.

In this study, we reviewed a large cohort of 599 patients diagnosed with locally advanced RC and treated with a standard treatment composed by neoadjuvant chemoradiotherapy (NACRT) and radical surgery. Pathoclinical factors, together with serum apoAI, apoB and apoB-to-apoAI ratio, were then analyzed on their association with patients’ liver-metastasis-free survival (LMFS). Cutoff values with the best prediction efficiency were established for the apolipoprotein-related indices.

## Methods

### Patient cohort

Clinical data of locally advanced RC (pretreatment clinical stage T3-4N0M0 and cT1-4N1-2M0) patients were extracted from the medical record database of the Sun Yat-sen University Cancer Center. The cases would be eligible for this study if they met the following criteria: (i) pathological diagnosis was made from Jan. 1st 2007 to Apr. 30th 2016; (ii) age at diagnosis was from 18 to 75 years old; (iii) Karnofsky performance score ≥ 80; (iv) treatment procedure consisted of NACRT and radical (R0) resection. And the cases would be excluded if they had: (i) any other prior malignancies; (ii) history of excessive drinking or drug abuse; (iii) treatment with monoclonal antibody; (iv) regular use of lipid-modulating agents, such as fibrates, niacins and statins; (v) distant metastasis during treatment.

### Diagnostic and staging work-up

Initial pathological diagnosis of rectal cancer was obtained through biopsy under rectoscope. Local extension and lymph node involvement at diagnosis were evaluated through pelvic magnetic resonance imaging (MRI) and endoscopic ultrasonography. Metastasis in distant organs such as liver were detected through thoracoabdominal computed tomography (CT) and further confirmed through positron emission tomography, if necessary. Specimens from radical resection were sent for pathologic examinations to assess tumor infiltration and differentiation, numbers of examined and involved lymph nodes, and tumor regression grade (TRG) after NACRT. The stages of each patient before treatment and after surgery were both made according to the TNM classification of the Union for International Cancer Control-American Joint Cancer Committee. The TRG criteria used in our hospital was the Mandard’s 5-tier grading system.

### Detection of serum biomarkers

Before treatment, the serum levels of apoAI and apoB were detected via the LABOSPECT 008 biochemistry system (Hitachi, Tokyo, Japan). And the serum levels of carcinoembryonic antigen (CEA) and carbohydrate antigen 19 − 9 (CA19-9) were detected via the E170 electrochemiluminescent immunoassay system (Roche Diagnostics, Tokyo, Japan). All detection were in accordance with the manufacturers’ instructions.

### Chemoradiotherapy and surgery

Radiotherapy was administered by using a three-dimensional conformal or intensified modulated radiation technique. The irradiation target of each patient was delineated according to the guidelines of the International Commission on Radiation Units and Measurements Reports 50 and 62. The prescribed doses for macroscopic tumor (containing primary lesion and metastatic lymph nodes) and high-risk (containing pararectal, presacral, obturator, internal and common iliac) lymphatic drainage regions were 50 and 46 Gy, respectively. The patients were irradiated with a linear accelerator delivering an 8-MV photon beam, for totally 25 fractions (1 fraction per day, 5 days per week). Chemotherapy was administered every 3 weeks, with a regimen consisting of oxaliplatin 130 mg/m^2^ on Day 1, plus capecitabine 1000 mg/m^2^ twice daily on Days 1–14. Totally 8 cycles of chemotherapy was prescribed, including 2–4 and 4–6 cycles in the neoadjuvant and adjuvant phases, respectively. Radical surgery was scheduled 8–12 weeks after radiotherapy, in accordance with the standard of total mesorectal excision.

### Follow-up

In the first 3 years after treatment, follow-up was performed every 3–6 months through outpatient interview. After the third year, follow-up was performed every 6–12 months, through outpatient interview or telephone. At each outpatient interview, the patients received a complete physical examination, thoraco-abdominal CT, pelvic MRI, serum CEA and CA19-9 tests. Rectoscope and whole-body bone scan were arranged annually. Follow-up lasted until death from rectal cancer (confirmed by death certificates) or Apr. 30th 2021, whichever came first.

### Endpoint definition

The primary endpoint in this study was LMFS, which was defined as the percentage of the patients surviving without LM over a given time period from pathologic diagnosis. The secondary endpoints included overall survival (OS) and other-metastasis-free survival (OMFS). The OS was defined as the percentage of the patients surviving over a given time period from diagnosis. And the OMFS was defined as the percentage of the patients who survived without metastasis other than LM, over a given time period from diagnosis.

### Variables and cutoff values

The variables for survival analysis included age, gender (male vs. female), tumor differentiation (poorly vs. moderately-well), distance to anal verge, pretreatment T stage (cT4 vs. cT3-1), pretreatment N stage (cN + vs. cN0), CEA, CA19-9, apoAI, apoB, apoB-to-apoAI ratio, active viral hepatitis (yes vs. no), TRG (5 − 3 s. 2 − 1), postsurgical T stage (pT4 vs. pT3-0), postsurgical N stage (pN + vs. pN0), and total chemotherapy cycle. The cutoff values of distance to anal verge was 5 (< 5 vs. ≥ 5) cm, which was the cutoff value for distal and proximal RC in the guidelines of the National Comprehensive Cancer Network. The cutoff value of chemotherapy cycle was 8 (< 8 vs. ≥ 8), which was proved by our previous work to influence distant metastasis rate of RC [12]. The best cutoff values of the rest continuous variables were the values achieving the maximum Youden indices in receiver operating characteristic (ROC) analysis.

### Survival analysis

The candidate predictors of the LMFS, OM and OMFS were first screened through univariate analysis based on the Kaplan-Meier approach. Survival difference between the patients grouped by each variable was tested by the log-rank test. The variables with significant difference were then entered into multivariate analysis based on the Cox proportional hazards model. The hazard ratio (HR) and 95% confidence interval (CI) was calculated for each variable. A variable was considered as independent predictors if it had a HR significantly different from the reference HR.

**Fig. 1:**
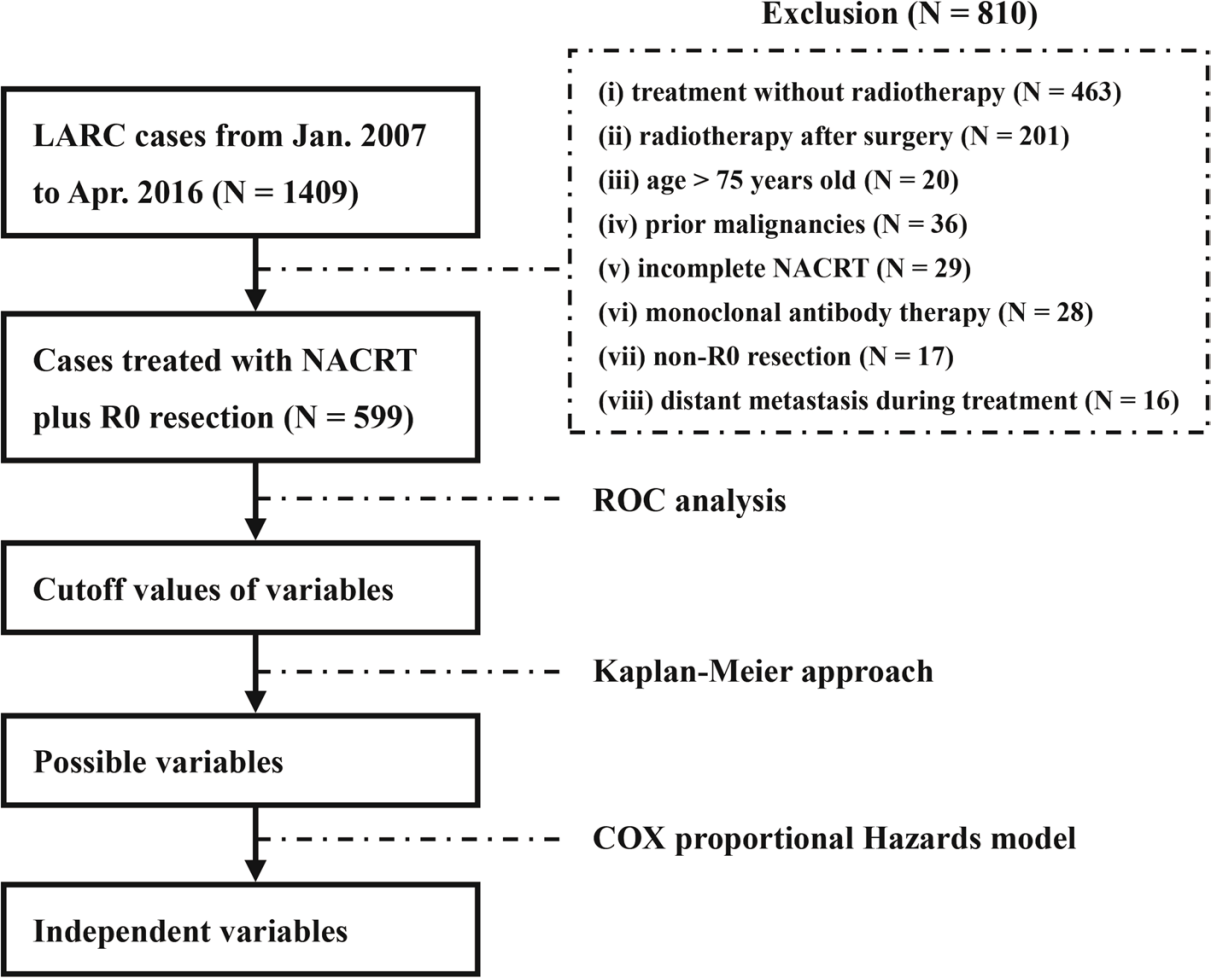
Analysis process of this study. Abbreviations: LARC, locally advanced rectal cancer; NACRT, neoadjuvant chemoradiotherapy; ROC, receiver operating characteristic

All the statistical analyses of this study were completed through the IBM SPSS Statistics 23.0 (IBM Corp., Armonk, NY, USA). The analysis process was summarized as Fig. 1. A difference with a two-sided *P* value of < 0.05 was regarded to have statistically significance.

## Results

### Baseline pathoclinical profiles

From Jan. 2007 to Apr. 2016, there were a total of 1409 consecutive patients diagnosed with untreated locally advanced RC in our hospital. Among them, 810 cases were excluded for: (i) treatment without radiotherapy (N = 463); (ii) radiotherapy after surgery (N = 201); (iii) age > 75 years old (N = 20); (iv) prior malignancies (N = 36); (v) incomplete NACRT (N = 29); (vi) monoclonal antibody therapy (N = 28); (vii) non-R0 resection (N = 17); (viii) distant metastasis during treatment (N = 16). Then 599 cases receiving NACRT followed by R0 resection were eligible for subsequent analyses. The baseline clinical characteristics of the study cohort were summarized in Table 1. Continuous and categorical data are presented as median (range) and number (percentage), respectively.

**Table 1:** Pretreatment pathoclinical characteristics of the 599 patients eligible for this study

Characteristic	Value
Age at diagnosis (years old)	56 (18–75)
No. of patients by gender	
Male	396 (66.1%)
Female	203 (33.9%)
No. of patients by tumor differentiation	
Low	63 (10.5%)
Moderate	458 (76.5%)
High	78 (13.0%)
Distance to anal verge (cm)	5.0 (1.0–15.0)
No. of patients by clinical T stage	
cT4b	26 (4.3%)
cT4a	249 (41.6%)
cT3	314 (52.4%)
cT2	10 (1.7%)
cT1	0 (0.0%)
No. of patients by clinical N stage	
cN2	213 (35.6%)
cN1	280 (46.7%)
cN0	106 (17.7%)
CEA (ng/ml)	4.2 (0.0-392.0)
CA19-9 (U/ml)	13.8 (0.0-985.6)
ApoAI (g/L)	1.19 (0.51–2.05)
ApoB (g/L)	0.90 (0.39–1.78)
ApoB-to-apoAI ratio	0.75 (0.23–1.92)
No. of patients by radiotherapy technique	
3DCRT	163 (27.2%)
IMRT	436 (72.8%)
No. of patients by TRG	
5	16 (2.7%)
4	107 (17.8%)
3	202 (33.7%)
2	137 (22.9%)
1	137 (22.9%)
No. of patients by pathological T stage	
pT4b	13 (2.2%)
pT4a	36 (6.0%)
pT3	240 (40.1%)
pT2	135 (22.5%)
pT1	24 (4.0%)
pT0	151 (25.2%)
No. of patients by pathologic N stage	
pN2	26 (4.3%)
pN1	103 (17.2%)
pN0	470 (78.5%)
Chemotherapy cycle	7 (4–10)
No. of patients by active viral hepatitis	
Yes	82 (13.7%)
No	517 (86.3%)

*Abbreviations: CEA *carcinoembryonic antigen; *CA19-9 *carbohydrate antigen 19 − 9; *apoAI *apolipoprotein A-I; *apoB *apolipoprotein B; *3DCRT *three-dimensional conformal radiation therapy; *IMRT*, intensified modulated radiation therapy; *TRG*, tumor regression grade

### Cutoff values of variables

The study cohort was followed up for a median time of 71 (range, 10–162) months. The number of patients lost to follow-up was 31 (5.2%). Death happened in 130 patients (21.7%), including 129 (21.5%) RC-related deaths. Recurrence happened in 33 (5.5%) patients. And distant metastases happened in 167 (27.9%) patients, including 134 (22.4%) lung, 104 (17.4%) liver, 17 (2.8%) bone, 7 (1.2%) brain, 7 (1.2%) peritoneum, 1 (0.2%) abdominal wall, and 1 (0.2%) cervical lymph node metastases. ROC analysis was performed for age, apoAI, apoB, apoB-to-apoAI ratio, CEA, and CA19-9 (supplementary materials, Table S1). The best cutoff values of these 6 variables for LM prediction were 67 (≥ 67 vs. < 67) years old, 1.22 (< 1.22 vs. ≥ 1.22) g/L, 1.06 (≥ 1.06 vs. < 1.06) g/L, 0.63 (≥ 0.63 vs. < 0.63), 6.1 (≥ 6.1 vs. < 6.1) ng/ml, and 26.3 (≥ 26.3 vs. < 26.3) U/ml.

### Patient survival

**Fig. 2:**
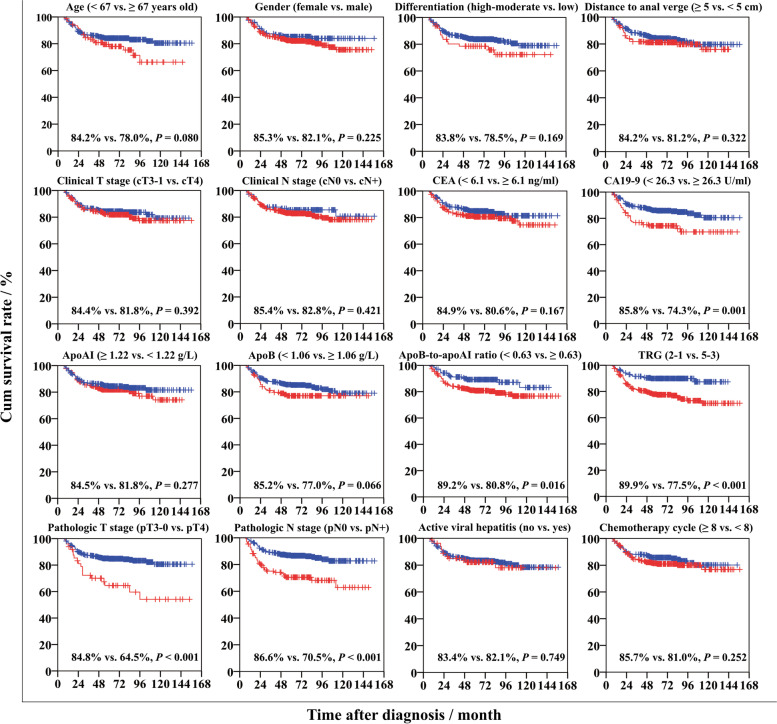
Liver-metastasis-free survival of the patients grouped by different pathoclinical factors. CA19-9 ≥ 26.3 U/ml, apoB-to-apoAI ratio ≥ 0.63, TRG 5 − 3, pT4 and pN + stage correlated with a decreased 5-year liver-metastasis-free survival. Abbreviations: CEA, carcinoembryonic antigen; CA19-9, carbohydrate antigen 19 − 9; apoAI, apolipoprotein A-I; apoB, apolipoprotein B; TRG, tumor regression grade

In univariate analysis, the patients with CA19-9 ≥ 26.3 U/ml, apoB-to-apoAI ratio ≥ 0.63, TRG 5 − 3, pT4 and pN + stage appeared to have a poorer 5-year LMFS (*P* values were 0.001, 0.016, < 0.001, < 0.001 and < 0.001; Fig. 2), respectively. The patients with age ≥ 67 years old, distance to anal verge < 5 cm, cT4 stage, CEA ≥ 6.1 ng/ml, CA19-9 ≥ 26.3 U/ml, TRG 5 − 3, pT4 and pN + stage had a poorer 5-year OS (*P* values were 0.004, 0.018, 0.026, 0.032, < 0.001, < 0.001, < 0.001 and < 0.001; Figure S1), respectively. And the patients with CEA ≥ 6.1 ng/ml, CA19-9 ≥ 26.3 U/ml, TRG 5 − 3, pT4 and pN + stage had a poorer 5-year OMFS (*P* values were < 0.001, < 0.001, < 0.001, 0.008 and < 0.001; Figure S2), respectively.

**Fig. 3:**
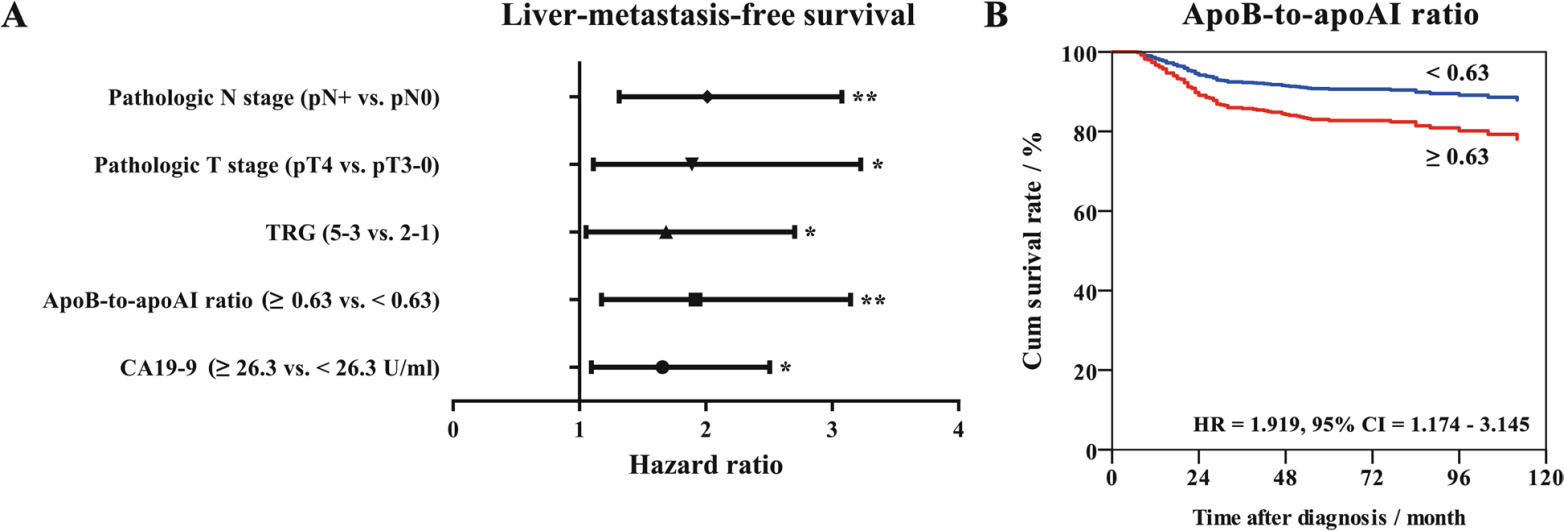
Multivariate survival analysis involving possible predictors of liver-metastasis-free survival. **A**: CA19-9 ≥ 26.3 U/ml, apoB-to-apoAI ratio ≥ 0.63, TRG 5 − 3, pT4 and pN + stage maintained to independently predict a decreased 5-year liver-metastasis-free survival. **B**: the liver-metastasis-free survival curves of the patients with different apoB-to-apoAI ratio were adjusted by the COX proportional hazards model. Abbreviations: CA19-9, carbohydrate antigen 19 − 9; apoAI, apolipoprotein A-I; apoB, apolipoprotein B; TRG, tumor regression grade; HR, hazard, ratio; CI, confidence interval. * *P* < 0.05, ** *P* < 0.01

Next, multivariate analyses were performed to evaluate the predictive independence of the possible predictors. As a result, CA19-9, apoB-to-apoAI ratio, TRG, pathologic T and N stages maintained to be independent predictors of LMFS (*P* values were 0.017, 0.009, 0.030, 0.019 and 0.001; Fig. 3 A), respectively. The adjusted HR for LMFS in the patients with apoB-to-apoAI ratio ≥ 0.63 was 1.919 (95% CI, 1.174–3.145; Fig. 3B). For OS, age, distance to anal verge, CA19-9, TRG, pathologic T and N stages maintained to be independent predictors (*P* values were 0.001, 0.001, 0.007, 0.003, 0.019 and 0.004), respectively. But clinical T stage and CEA failed to exhibit predictive independence (Figure S3A). And for OMFS, CA19-9, TRG, pathologic T and N stages maintained to be independent predictors (*P* values were 0.001, 0.020, 0.010, 0.036 and 0.004), respectively. CEA failed to exhibit predictive independence (Figure S3B).

In addition, the candidate predictors were compared between the patients with apoB-to-apoAI ratios of < 0.63 and ≥ 0.63. Unbalanced distribution was seen in none of the factors (Table S2).

## Discussion

LM is frequently seen in locally advanced RC. In this study, 17.4% of the patients were finally observed to undergo LM, after a median follow-up time of nearly 6 years. This figure was similar to prior studies [6, 13]. To select predictors of LM, a series of pathoclinical factors were analyzed for their association with LM risk. The results indicated that CA19-9 ≥ 26.3 U/ml, apoB-to-apoAI ratio ≥ 0.63, TRG 5 − 3, pT4 and pN + stage correlated independently with a decreased 5-year LMFS. The HRs were 1.656 (95% CI, 1.094–2.506), 1.919 (95% CI, 1.174–3.145), 1.686 (95% CI, 1.053–2.703), 1.890 (95% CI, 1.110–3.226) and 2.012 (95% CI, 1.314–2.077), respectively. The CA19-9, TRG, and pathologic T and N stages were all balanced between the patients with different apoB-to-apoAI ratios. It further supported apoB-to-apoAI ratio as an independent predictor of LM.

Also, the same factors were analyzed for their association with risk of metastases to other organs. Of those, CA19-9 ≥ 26.3 U/ml, TRG 5 − 3, pT4 and pN + stages correlated independently with a decreased 5-year OMFS. The HRs were 1.631 (95% CI, 1.079–2.469), 1.848 (95% CI, 1.155–2.959), 1.764 (95% CI, 1.038–2.994) and 1.848 (95% CI, 1.209–2.825), respectively. But apoB-to-apoAI exhibited no correlation with patients’ OMFS. Namely, apoB-to-apoAI was a factor specifically predicting LM from RC. Since pathologic TNM stage and TRG could only be determined after surgery, apoB-to-apoAI had a superiority in helping clinicians to evaluate individual risk of LM at an early time. Additionally, all the predictors of LMFS also emerged as independent predictors of OS, except apoB-to-apoAI. The possible reason was that metastases in liver and other organs both contributed to cancer-related death.

Mounting evidences connect lipid metabolism to biological behaviors of cancer cells, including proliferation, apoptosis, invasion and migration. A key mechanism is the lipid raft comprising of cholesterol and sphingolipid on cell membrane. This microdomain functions as a signal transduction platform and selectively recruits receptors, adhesion molecules and signaling molecules. ApoAI and apoB are both cholesterol transporters in blood circulation but have different destinations. ApoAI transports excess cholesterol to liver cells where cholesterol is transformed into bile acids. Oppositely, apoB is responsible for cholesterol accumulation in peripheral tissue and tumor cells [7, 14, 15]. It confers these two apolipoproteins different roles in regulating cancer pathophysiology.

ApoAI is now believed to have anti-tumor activities. Laboratory studies indicated that apoAI exerted inhibitory effects on growth and metastasis of cancer cells, both in vitro and in vivo. Besides, it could modulate tumor microenvironment, including decrease of immune escape-related cells (myeloid-derived suppressor cells, etc.), increase of tumor killer cells (CD8 + T-lymphocytes, etc.), induction of macrophage M2/M1 phenotype shift, and suppression of VEGF-mediated angiogenesis [16]. In clinical studies, low level of serum apoAI was associated with poor OS of some solid tumors, including lung, renal, and esophageal carcinoma [17–19]. One previous study by us showed that low serum apoAI predict a poor OS as well as increased distant metastases in nasopharyngeal carcinoma [20]. Though relative reports were not as many as apoAI, apoB is considered to have tumor-promoting effects. Liu et al. found a correlation between polymorphisms of apoB gene and risk of breast cancer [21]. Cedó et al. further found that apoB stimulated growth of estrogen receptor-positive breast cancer cells, via transporting 27-hydroxycholesterol [22].

Recently, the prognostic implications of serum apoAI and apoB in colorectal cancer have been gradually revealed. Ye et al. and Chen et al. reported that serum apoAI and apoB as positive and negative predictors of overall and progression-free survivals in colorectal cancer, respectively [9, 10]. Another previous study by us showed that low serum apoAI led to a bad NACRT-response in RC [23]. Some studies combined apoAI and apoB into a more powerful index, apoB-to-apoAI ratio. Sirniö et al. and Yang et al. found high apoB-to-apoAI ratio as a risk factor of cancer-related death, in both non-metastatic and metastatic colorectal cancer [11, 24]. As is known, apoAI and apoB are mainly synthesized and released by liver cells [14]. Combining their synthesis site and regulatory functions in cancer, it is not hard to understand their abilities in predicting LM.

Lipid-modulating drugs have already been put into clinical application for a long time. Some of them are able to affect serum levels of apolipoproteins. For example, statins and niacins could elevate serum apoAI level [25, 26]. Fibrate could increase serum apoAI and decrease serum apoB simultaneously [27]. Hence, the results of this study provided a possibility to reduce LM risk by using these drugs. On the other side, apoAI mimetic peptides could now be synthesized artificially, including D-4 F, L-4 F, L-5 F and Tg6F. These mimetics were found to reduce metastasis of colorectal cancer in animal models and expected to become new agents for managing LM [7]. Moreover, recombinant apoB could be used as a carrier of anti-tumor drugs, such as chemotherapy agents and small interfering RNA targeting oncogenes. It might be regarded as another therapeutic choice for LM from RC [15].

As far as we know, this study presented apoB-to-apoAI ratio as an independent predictor of LM from RC for the first time. The following advantages made its results quite reliable. First, this study analyzed a large cohort of 599 locally advanced RC patients. Second, the patients were treated uniformly with NACRT plus surgery, the current mainstream treatment. Third, all the patients were followed up for a sufficient time of 5 years. Fourth, as many known factors of RC were involved as possible for screening. Yet, selection biases might not completely be avoided in this study, due to its retrospective, single-institutional nature. So we suggested the results be validated in a prospective cohort, or a cohort from other institution.

## Conclusions

Pretreatment level of serum apoB-to-apoAI ratio was associated negatively with the 5-year LMFS of locally advanced RC patients. It could be considered as a new predictive biomarker and potential therapeutic target for LM from RC.

## Supplementary Information

**Additional file 1.**

## Data Availability

The datasets used and/or analyzed during the current study are available from the corresponding author on reasonable request.

## Abbreviations

RC: rectal cancer
LM: liver metastasis
apoAI: apolipoprotein A-I
apoB: apolipoprotein B
NACRT: neoadjuvant chemoradiotherapy
LMFS: liver-metastasis-free survival
MRI: magnetic resonance imaging
CT: computed tomography
CEA: carcinoembryonic antigen
CA19-9: carbohydrate antigen 19-9
TRG: tumor regression grade
OS: overall survival
OMFS: other-metastasis-free survival
ROC: receiver operating characteristic
HR: hazard ratio
CI: confidence interval
