# Supplementary material for: Serum apolipoprotein B to apolipoprotein A-I ratio is an independent predictor of liver metastasis from locally advanced rectal cancer in patients receiving neoadjuvant chemoradiotherapy plus surgery
Source: BMC Cancer. 2022 Jan 3;22:7. doi: 10.1186/s12885-021-09101-y (PMC8722169; doi:10.1186/s12885-021-09101-y)

**Table S1 The best cutoff values of continuous variables for predicting liver metastasis.**

| **Variable** | **Cutoff value** | **Sensitivity** | **Specificity** | **Youden index** |
| --- | --- | --- | --- | --- |
| Age (years old) | 67 (≥ 67 vs. < 67) | 21.2% | 85.9% | 0.071 |
| ApoAI (g/L) | 1.22 (< 1.22 vs. ≥ 1.22) | 50.0% | 55.6% | 0.056 |
| ApoB (g/L) | 1.06 (≥ 1.06 vs. < 1.06) | 30.8% | 77.4% | 0.082 |
| ApoB-to-apoAI ratio | 0.63 (≥ 0.63 vs. < 0.63) | 80.8% | 31.3% | 0.121 |
| CEA (ng/ml) | 6.1 (≥ 6.1 vs. < 6.1) | 44.2% | 63.4% | 0.076 |
| CA19-9 (U/ml) | 26.3 (≥ 26.3 vs. < 26.3) | 34.6% | 80.0% | 0.146 |

Abbreviations: CEA, carcinoembryonic antigen; CA19-9, carbohydrate antigen 19-9; apoAI, apolipoprotein A-I; apoB, apolipoprotein B.

**Table S2 Candidate predictors in patients with different serum apolipoprotein B to apolipoprotein A-I ratios.**

|  | **ApoB-to-apoAI ratio < 0.63 (N = 175)** | **ApoB-to-apoAI ratio ≥ 0.63 (N = 424)** | **Chi-square** | ***P* value** |
| --- | --- | --- | --- | --- |
| **Age (years old)** |  |  |  |  |
| **< 67** | 148 (84.6%) | 359 (84.7%) | 0.001 | 0.976 |
| **≥ 67** | 27 (15.4%) | 65 (15.3%) |  |  |
| **Gender** |  |  |  |  |
| **Female** | 69 (39.4%) | 134 (31.6%) | 3.385 | 0.066 |
| **Male** | 106 (60.6%) | 290 (68.4%) |  |  |
| **Differentiation** |  |  |  |  |
| **High-moderate** | 156 (89.1%) | 380 (89.6%) | 0.030 | 0.862 |
| **Low** | 19 (10.9%) | 44 (10.4%) |  |  |
| **Distance to anal verge (cm)** |  |  |  |  |
| **≥ 5** | 110 (62.9%) | 291 (68.6%) | 1.867 | 0.172 |
| **< 5** | 65 (37.1%) | 133 (31.4%) |  |  |
| **Clinical T stage** |  |  |  |  |
| **cT3-1** | 93 (53.1%) | 231 (54.5%) | 0.089 | 0.765 |
| **cT4** | 82 (46.9%) | 193 (45.5%) |  |  |
| **Clinical N stage** |  |  |  |  |
| **cN0** | 31 (17.7%) | 75 (17.7%) | 0.000 | 0.994 |
| **cN+** | 144 (82.3%) | 349 (82.3%) |  |  |
| **CEA (ng/ml)** |  |  |  |  |
| **< 6.1** | 108 (61.7%) | 262 (61.8%) | 0.000 | 0.986 |
| **≥ 6.1** | 67 (38.3%) | 162 (38.2%) |  |  |
| **CA19-9 (U/ml)** |  |  |  |  |
| **< 26.3** | 134 (76.6%) | 329 (77.6%) | 0.074 | 0.786 |
| **≥ 26.3** | 41 (23.4%) | 95 (22.4%) |  |  |
| **TRG** |  |  |  |  |
| **2-1** | 89 (50.9%) | 185 (43.6%) | 2.605 | 0.106 |
| **5-3** | 86 (49.1%) | 239 (56.4%) |  |  |
| **Pathologic T stage** |  |  |  |  |
| **pT3-0** | 162 (92.6%) | 388 (91.5%) | 0.186 | 0.666 |
| **pT4** | 13 (7.4%) | 36 (8.5%) |  |  |
| **Pathologic N stage** |  |  |  |  |
| **pN0** | 131 (74.9%) | 339 (80.0%) | 1.903 | 0.168 |
| **pN+** | 44 (25.1%) | 85 (20.0%) |  |  |
| **Active viral hepatitis** |  |  |  |  |
| **No** | 149 (85.1%) | 368 (86.8%) | 0.285 | 0.593 |
| **Yes** | 26 (14.9%) | 56 (13.2%) |  |  |
| **Chemotherapy cycle** |  |  |  |  |
| **≥ 8** | 88 (50.3%) | 194 (45.8%) | 1.021 | 0.312 |
| **< 8** | 87 (49.7%) | 230 (54.2%) |  |  |

Abbreviations: apoB, apolipoprotein B; apoAI, apolipoprotein A-I; CEA, carcinoembryonic antigen; CA19-9, carbohydrate antigen 19-9; TRG, tumor regression grade.

**Figure S1 Overall survival of the patients grouped by different pathoclinical factors.** Age ≥ 67 years old, distance to anal verge < 5 cm, cT4 stage, CEA ≥ 6.1 ng/ml, CA19-9 ≥ 26.3 U/ml, TRG 5-3, pT4 and pN+ stage correlated with a decreased 5-year overall survival. Abbreviations: CEA, carcinoembryonic antigen; CA19-9, carbohydrate antigen 19-9; apoAI, apolipoprotein A-I; apoB, apolipoprotein B; TRG, tumor regression grade.


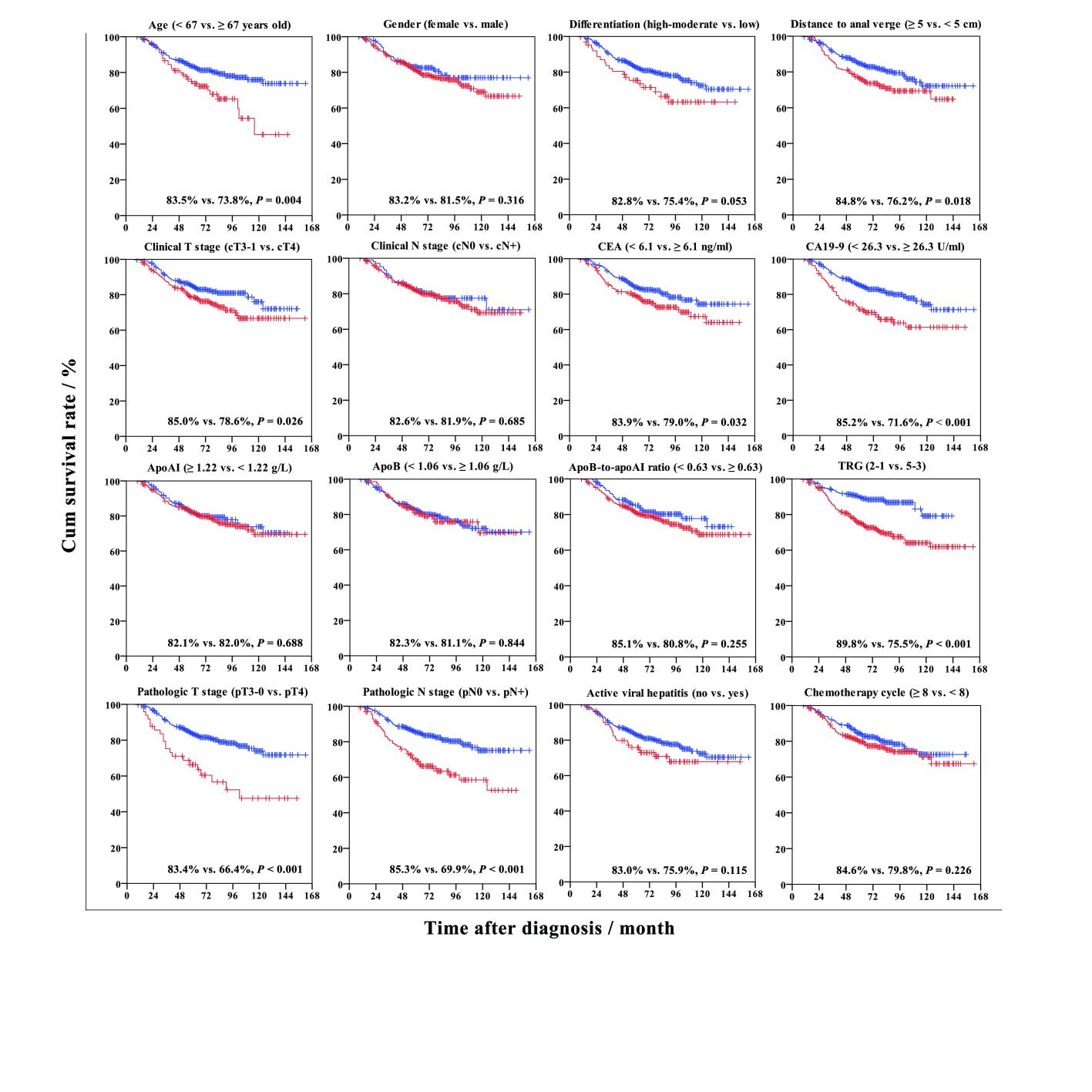


**Figure S2 Other-metastasis-free survival of the patients grouped by different pathoclinical factors.** CEA ≥ 6.1 ng/ml, CA19-9 ≥ 26.3 U/ml, TRG 5-3, pT4 and pN+ stage correlated with a decreased 5-year other-metastasis-free survival. Abbreviations: CEA, carcinoembryonic antigen; CA19-9, carbohydrate antigen 19-9; apoAI, apolipoprotein A-I; apoB, apolipoprotein B; TRG, tumor regression grade.


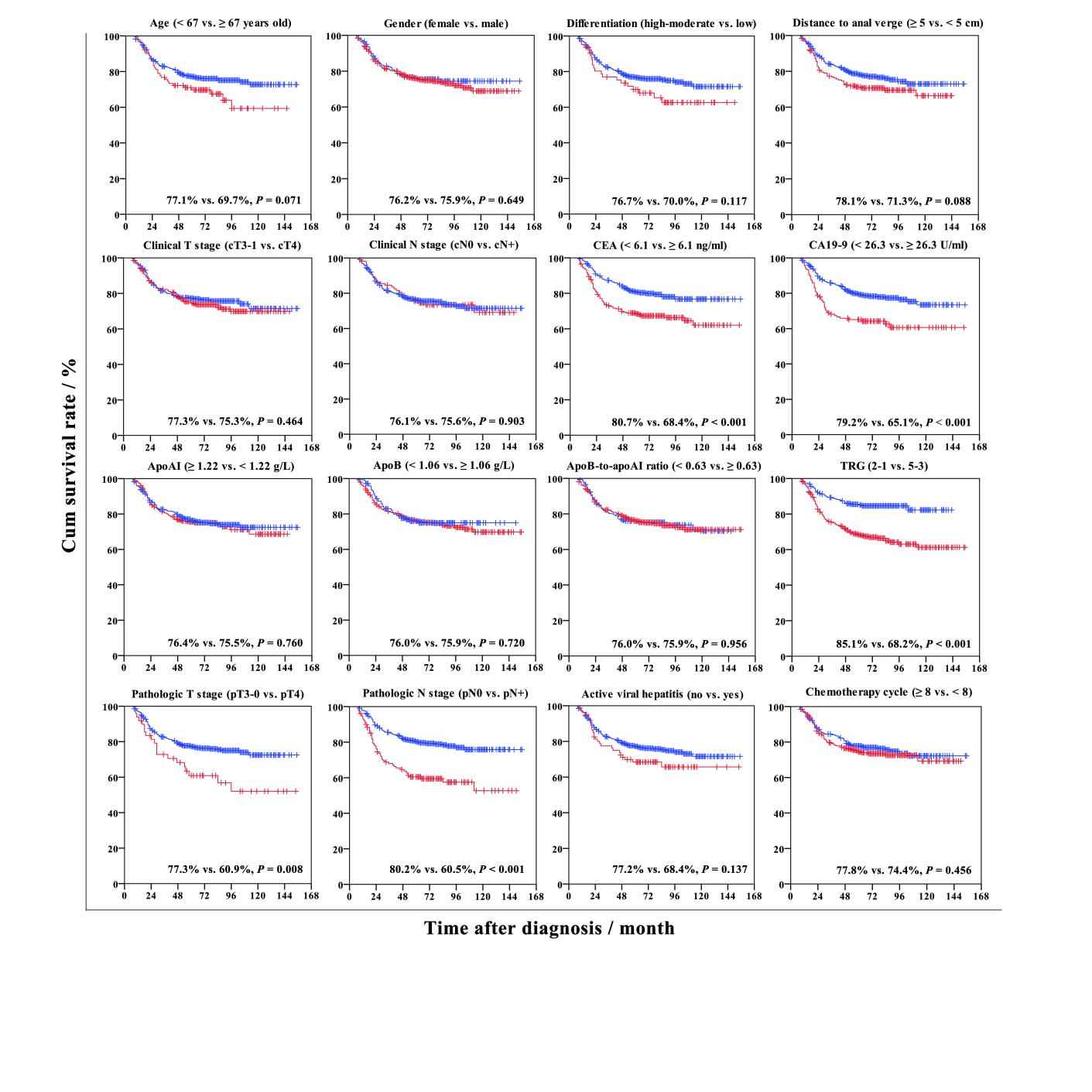


**Figure S3 Multivariate survival analysis involving possible predictors of overall and other-metastasis-free survivals. A:** age ≥ 67 years old, distance to anal verge < 5 cm, CA19-9 ≥ 26.3 U/ml, TRG 5-3, pT4 and pN+ stage maintained to independently predict a decreased 5-year overall survival. B: CA19-9 ≥ 26.3 U/ml, TRG 5-3, pT4 and pN+ stage maintained to independently predict a decreased 5-year other-metastasis-free survival. Abbreviations: CEA, carcinoembryonic antigen; CA19-9, carbohydrate antigen 19-9; TRG, tumor regression grade. * *P* < 0.05, ** *P* < 0.01.


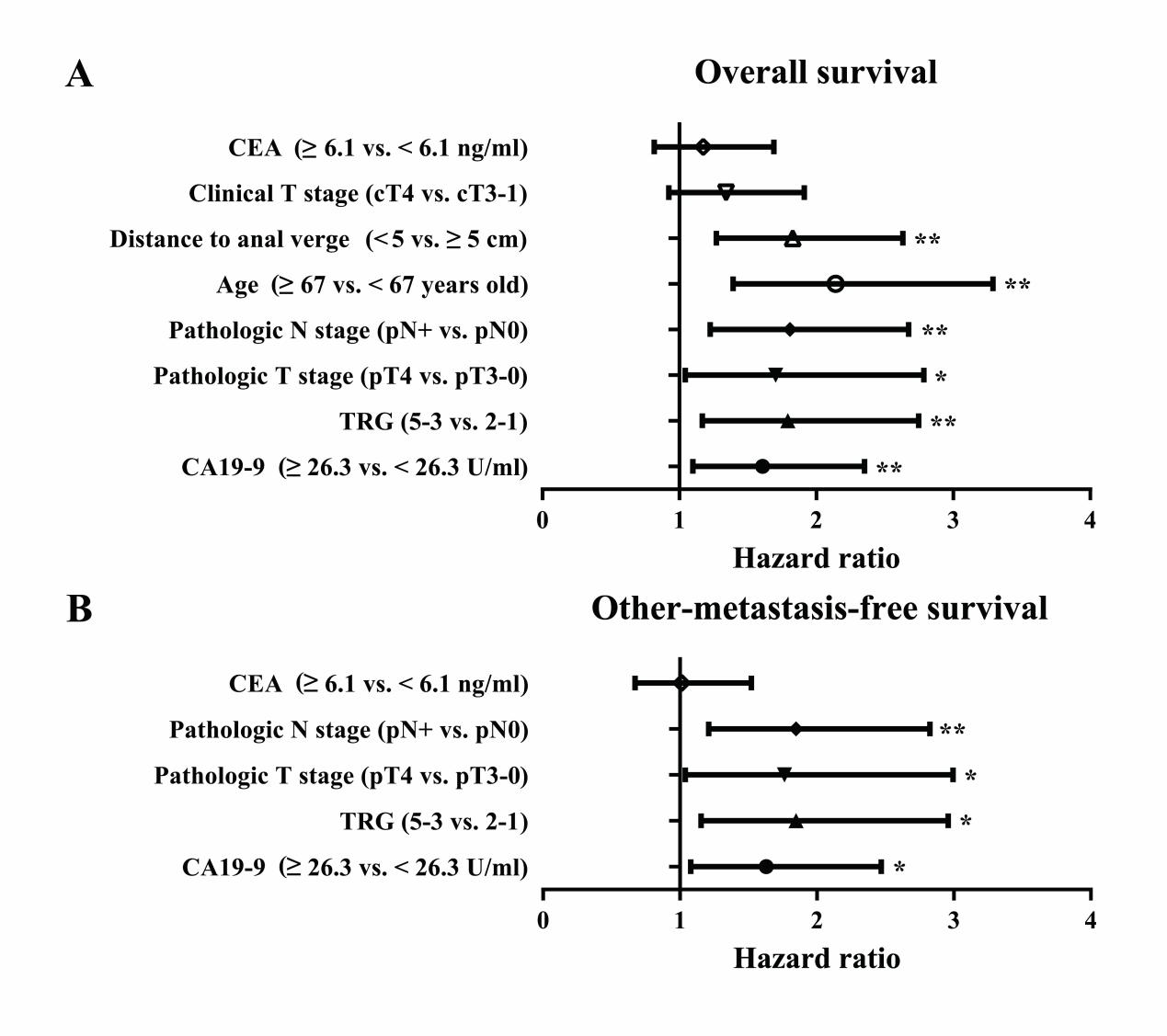

Supplement: Supplementary file 1 — Additional file 1. [file 12885_2021_9101_MOESM1_ESM.docx]
